# Utilization of obstetric analgesia for labor pain management and associated factors among obstetric care providers working in hospitals of South East and South Central Ethiopia, 2023

**DOI:** 10.1186/s12884-026-09310-y

**Published:** 2026-05-22

**Authors:** Sintayehu Solomon Kena, Nathan Desalegn, Bitew Mekonnen, Hiwot Tadesse, Tewodros Getachew Tsegaye, Anteneh Gezahgn Wordofa, Birhanemeskel Damtew Legesse, Betelhem Melkamu Wachefo

**Affiliations:** 1 Department of Midwifery, College of Medicine and Health Sciences, Hawassa University, Hawassa, Ethiopia; 2 School of Nursing, College of Medicine and Health Sciences, Arba Minch University, Arba Minch, Ethiopia; 3 School of Nursing and Midwifery, College of Health and Medical Sciences, Haramaya University, Harar, Ethiopia; 4 Dilla University General Hospital, Dilla, Ethiopia; 5 Department of Midwifery, College of Health Sciences and Medicine, Wachemo University, Durame, Ethiopia

**Keywords:** Pharmacologic method, Non-pharmacologic method, Health care providers, Pain

## Abstract

**Introduction:**

The pain a woman feels during labor and delivery is unique to her, and obstetric analgesia should be provided upon request by mothers. In low-income countries, most women continue to endure painful labor despite the availability of methods. The factor for utilization remains inconsistent in previous studies, and they did not assess client factors as a reason for the nonuse of obstetric analgesia, and the majority of them excluded private hospitals. The study aimed to assess the magnitude of utilizations of obstetrics analgesia and associated factors among Obstetrics care providers.

**Methods:**

A facility-based – cross-sectional study was conducted from March 10 to April 20, 2023, among obstetric care providers in hospitals in the West Arsi and Arsi zones. A structured self-administered questionnaire was used to collect the data. The data were entered into Epi data statistical software version 3.1 and exported to SPSS window version 26 for analysis. Descriptive statistical analysis was performed. Bivariate and multivariable logistic regression analyses were used to identify factors associated with the use of obstetric analgesia. A P- value < 0.05 with a 95% CI was considered to indicate an association.

**Results:**

Out of the 421 participants enrolled in the study, 52.3% (95% CI: 47.46, 57.14) reported using obstetric analgesia in the previous month. Factors such as sex [AOR: 1.86 (95% CI: 1.14, 3.04)], working in a general hospital [AOR: 2.86 (95% CI: 1.41, 5.80)], working in a primary hospital [AOR: 3.89 (95% CI: 1.85, 8.17)], having adequate knowledge [AOR: 1.94 (95% CI: 1.05, 3.57)], and having a favorable attitude [AOR: 6.92 (95% CI: 3.97, 12.06)] were significantly associated the use of obstetric analgesia.

**Conclusion and recommendation:**

The use of obstetric analgesia in this study was low compared to previous studies. Creating a suitable environment by increasing health professionals’ knowledge and attitudes regarding labor management methods is necessary.

**Supplementary Information:**

The online version contains supplementary material available at 10.1186/s12884-026-09310-y.

## Background

Labor pain is considered an “excellent model for acute pain” [1]. Unlike other acute pain that is usually associated with injury or pathology, they are part of a normal physiological process [2]. The American College of Obstetricians and Gynecologists (ACOG) confirmed the severity of labor pain by stating that no other condition was as severe as labor pain [3]. There are two elements of labor pain: visceral and somatic. The Visceral Pain occurs during the first stage of labor and is associated with tension placed on the cervix, causing it to dilate. Somatic pain appears at the end of the first stage and continues into the second stage. It is caused by the force applied to the vaginal part of the cervix, the vagina, and the perineum [4].

Pain management during labor entails several activities, including pain assessment, pain control, and pain relief. Therefore, obstetric care providers must use safe and effective interventions [5]. A variety of pharmacological and non-pharmacological interventions have been developed to manage labor pain, including opioids, nonopioids, epidural analgesia, spinal-epidural analgesia, inhalation agents, pudendal blocks, transcutaneous electrical nerve stimulation, massage, acupuncture, immersion in water, yoga, music therapy, biofeedback, continuous support, positioning, ambulation, hypnosis, and breathing techniques [6, 7].

Several organizations, including the ACOG and the American Society of Anesthesiologists (ASA), have stated that it is unacceptable for clients to suffer untreated severe pain under the care of a physician and obstetric analgesia should be offered upon request by mothers [3, 8]. Additionally, the National Institute for Health and Care Excellence (NICE) of the UK recommends that women be educated about the options and availability of effective analgesia during labor to ensure that they receive the best pain relief [9]. The Ethiopian Food, Medicine, and Health Care Administration and Control Authority (FMHACA) has developed and implemented standard treatment guidelines, which include administering analgesics and anesthetics to pregnant mothers without adversely affecting their condition or that of the fetus [10].

Labor pain and pain relief methods are major concerns for childbearing women and their families, with major implications for the course, quality, outcome, and cost of intrapartum care [11]. The worst pain a woman experiences is during labor [12]. Previously conducted researches indicate the magnitude of obstetrics analgesia utilization among obstetrics care providers that ranges from 38.9 to 93.5% [13, 14]. When a woman experiences extreme pain during labor, she experiences emotional discomfort, depression, and anxiety.

Moreover, psychosocial aspects such as cultural beliefs, support from family, marital and social status, and past experiences of difficult labor can also play a role in increasing the pain experienced during childbirth. Furthermore, these factors can influence the mother’s health and her bonding with the child, with potential effects lasting both in the short and long term [15].

In addition to fear of labor pain, a lack of appropriate labor pain management causes laboring mothers to become concerned about or request cesarean sections, which exposes them to financial hardships [16]. It is important to note, however, that complete pain relief may not always equate to a more satisfying birth experience [17].

The provision of pain relief during labor is ignored despite the need, benefits, and drawbacks of pain relief options, particularly pharmacological options [6]. The main factors affecting care providers’ use of labor analgesics in developing countries are drug availability, healthcare delivery systems, and healthcare providers’ knowledge, attitudes, and skills to provide labor analgesia [7]. The ACOG recommends that maternal request be a sufficient medical indication for pain relief during labor, unless there is a medical contraindication [3]. In Contrast to being an important component of the Ethiopian Federal Minister of Health (EFMOH) efforts to improve the quality of maternal health services available to Ethiopians, labor pain management methods are not widely used [18].

Despite national recommendations for obstetric analgesia use during labor, little is known about its utilization by care providers in the study area. This study aimed to assess the magnitude of obstetric analgesia utilization and associated factors in the study area.

## Method and materials

### Study design and setting

A facility-based cross sectional study was conducted in hospitals in the West Arsi and Arsi Zones from March 10 to April 20, 2023. The West Arsi and Arsi zones are two of the 20 administrative zones found in the Oromia regional state in Southwest and Southeast Ethiopia. The central city for the west Arsi zone is Shashemene, located 250 km from Addis Ababa, while Asella is the central city for the Arsi zone, located 175 km from Addis Ababa. The West Arsi zone has seven public hospitals and four private hospitals, while the Arsi zone has eight public hospitals and two private hospitals. In total, there were twenty one hospitals in the two zones, of which fifteen were public hospitals and six were private hospitals.

### Source population

All obstetric care providers in hospitals in the West Arsi and Arsi Zones of the Oromia region composed the source population. The study population included randomly selected obstetric care providers working in the hospital of West Arsi and Arsi Zone during the study period.

### Inclusion criteria and exclusion criteria

Obstetric care providers working in the hospitals of West Arsi and Arsi Zone during the study period were included in the study, while those absent due to annual and sick leave during the study period were excluded from the study.

### Sample size determination

The sample size was determined by using a single population proportion formula.

by considering the following assumptions: n = sample size, p = proportion of obstetric analgesia utilization from study performed in West Shewa 46% [19], Z = standard normal distribution curve value for 95% confidence level with the value of 1.96, d = margin of error to be tolerated (d = 0.05). By using this formula, the sample size become 382. By adding 10% non-response rate the final sample size for the study become 421 obstetric care providers.

### Sampling technique and sampling procedure

**Fig. 1:**
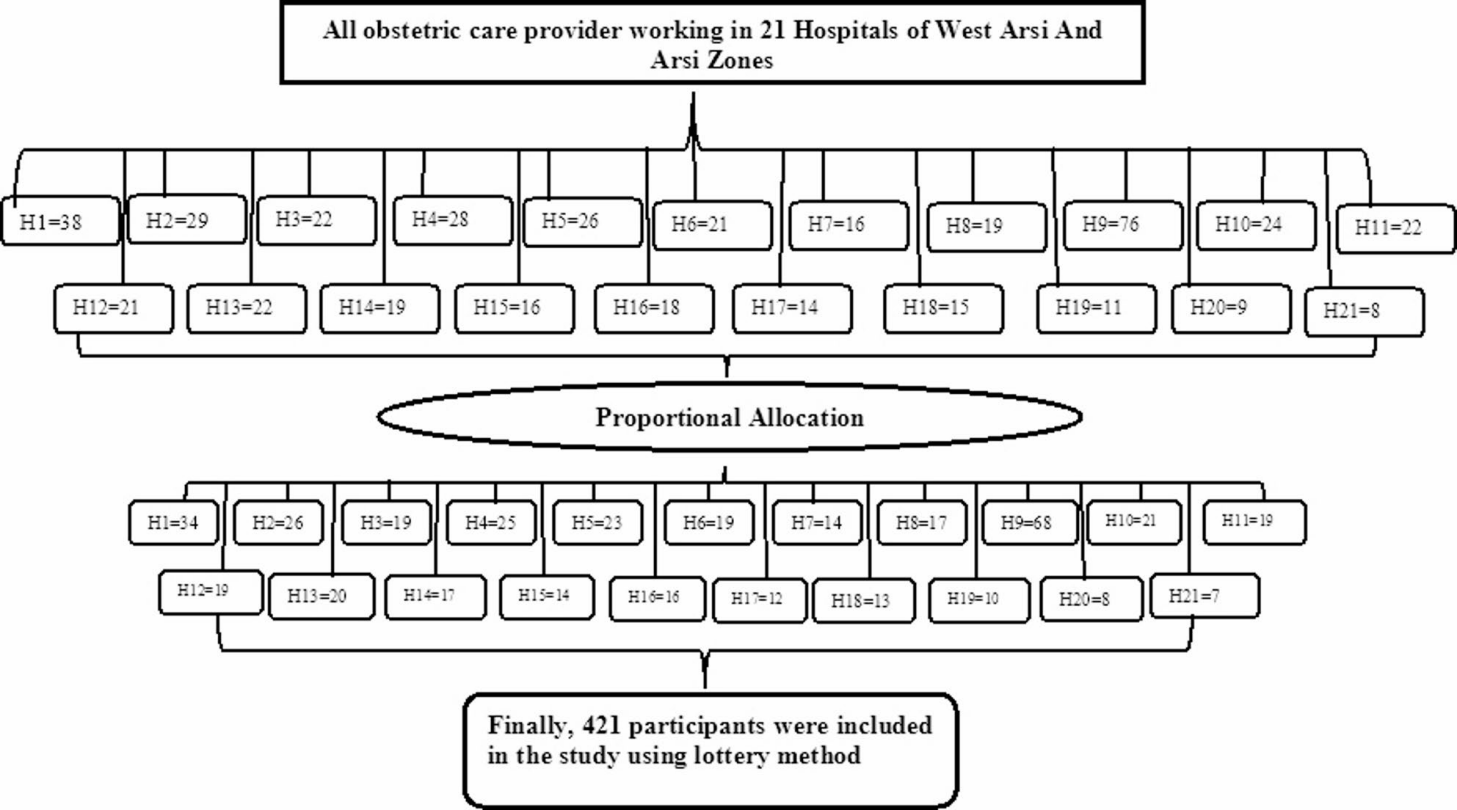
Diagrammatic presentation of sampling procedure of obstetric care providers working in hospitals of West Arsi and Arsi Zones, Oromia region, Ethiopia, 2023. H1- Shashemene comprehensive and Specialized Hospitals, H2- Melka oda General Hospital, H3- Negele Arsi primary Hospital, H4- Dodola General Hospitals, H5- Gambo General Hospital, H6- Loke primary Hospital, H7- Feya primary Hospital, H8- Negele Arsi General Hospital and Medical College, H9- Arsi university Referral and Teaching Hospital, H10- Bokoji primary Hospital, H11- Abomsa Primary Hospital, H12- Gobessa primary Hospital, H13- Kersa Primary Hospital, H14- Robe dida Primary Hospital, H15- Rohobot General Hospital, H16- Kokosa Primary Hospital, H17- Kula Primary Hospital, H18- Chancho Primary Hospital, H19- Dr. Firaol primary Hospital, H20 Chilalo Shade Hospital, H21 Essa Hospital

All hospitals in the West Arsi and Arsi Zones were included in the study. The study included; Shashemene Comprehensive and Specialized Hospital, Melka oda General Hospital, Negele Arsi primary Hospital, Dodola General Hospital, Gambo General Hospital, Loke Primary Hospital, Feya Primary Hospital, Negele Arsi General Hospital and Medical College, Kokosa Primary Hospital, Essa Primary Hospital, Kula Primary Hospital, Chancho Primary Hospital, Dr. Firaol Primary Hospital, Arsi University Referral and Teaching Hospital, Bokoji Primary Hospital, Abomsa Primary Hospital, Gobessa Primary Hospital, Kersa Primary Hospital, Robe Dida Primary Hospital, Chilalo shade Primary Hospital, and Rohobot General Hospital. The total sample size was proportionally allocated to each hospital by reviewing the number of obstetric care providers working there and the final study participants to be included were selected randomly through the lottery method, using the staff list as a sampling frame (Fig. 1).

### Data collection tool

The data were collected using a structured, pre tested, and self-administered questionnaire adapted from different studies [19–22]. The questionnaire was developed in English for all respondents to understand. It consisted of four essential components related to obstetric analgesia utilization in labor pain management, including providers’ sociodemographic characteristics, individual-related factors, facility-related factors, and client-related questions. The knowledge-related questions comprise fourteen items, and the attitude-related question comprises ten items with five possible options on a Likert scale (strongly disagree, disagree, undecided, agree, and strongly agree), and all statements were affirmatively stated. Cronbach’s Alpha test was calculated using SPSS window version 26 to test the internal consistency of the items, and the Cronbach’s Alpha test for the item was 0.835, which is considered reliable.

### Data collection procedures

Twelve BSc holding midwives were recruited as data facilitators, and two MSc holding nurses were recruited for supervision. The purpose of the study and the importance of their involvement were explained to the respondents, and those who volunteered participated in the self-administered questionnaires.

### Study variables

#### Dependent variable

Utilization of obstetric analgesia for labour pain management.

#### Independent variable

##### Socio-demographic characteristic

Age, Sex, Religion, Clinical Experience, Profession, and Level of Hospital.

##### Facility related factors

Availability of labor analgesia and Equipment, Protocol and Guideline, Number of Skilled providers, and Cost of analgesic drug.

##### Individual related factors

Attitude of care providers, Knowledge of care providers, Training, having heard of WHO pain ladder, Expectation of Labour pain, Level of Qualification, and Allowing companion.

##### Client related factors

Maternal Refusal.

### Operational definition

#### Utilization of obstetric analgesia

Obstetric care providers were considered to have utilized obstetric analgesia if they reported using any form of pharmacological or non-pharmacological method for labor pain management at least once during the past month prior to data collection [19].

#### Obstetric care providers

Skilled health professionals who were giving maternal care service (i.e., Midwives, Nurses, Health officers, General Practionaire, Residents, obstetrician and gynecologist, IESO, Anesthesiologists, Anesthetists).

#### Adequate knowledge

Obstetric care providers who answered more than or equal to the mean knowledge of obstetric analgesia related questions were considered to have adequate knowledge of obstetric analgesia, while those who answered less than the mean value were considered to have inadequate knowledge of obstetric analgesia [19–21].

#### Favorable attitudes

Obstetric care providers who answered greater than or equal to the mean value for attitudes related questions were considered to have favorable attitudes toward the use of obstetric analgesia, while those who answered less than the mean value were considered to have unfavorable attitudes toward the use of obstetric analgesia [19].

### Data quality control

To ensure the quality of the data, a pretest was conducted on 5% of the total sample size. Findings and experiences from the pretest were used to modify and refine the data collection tools. Training was provided to the data collectors and supervisors on the study objective, confidentiality of information, and data collection techniques. The completeness of the data was checked for missing values by the supervisors.

### Data processing and analysis

The data were coded, cleaned, and entered into Epi data statistical software version 3.1 and then exported to SPSS window version 26 for further analysis. Descriptive statistical analysis was used to summarize the data.

Bivariate regression models were first built for each explanatory variable with the outcome variable. Variables with a p-value < 0.25 in bivariate regression were fitted into multivariable logistic regression models for analysis. Multivariate logistic regression analysis was conducted to control for confounders and identify association between each explanatory variable and the outcome variable. Variables with a p-value < 0.05 with a 95% confidence interval in the final model were considered as having a significant association with the use of obstetric analgesia.

The Hosmer and Lemeshow statistical test were used to assess model fitness, and the results indicated a good fit (X^2^ = 2.07, df = 8, *p* = 0.978). The variance inflation factor (VIF) test was used to assess multicollinearity in the regression model, and all the VIF values were below 10, indicating no multicollinearity issues.

## Results

### Sociodemographic characteristics of study participants

Out of the 421 participants enrolled in the study, 413 completed and returned a response, yielding a response rate of 98.1%. The median age of the participants was 30 years (IQR = 27.5–33), and the median clinical experience of the participants was 5 years (IQR = 2–7). Of the respondents, 50.8% were male, and 41.6% were midwife professionals (Table 1).

**Table 1:** Sociodemographic characteristics of obstetric care providers in hospitals of West Arsi and Arsi zones, Oromia region, Ethiopia, 2023

Variables	Category	Frequency	Percent
Age	21–30	229	55.4%
	31–40	153	37.0%
	41–50	26	6.3%
	51–60	5	1.2%
Sex	Male	210	50.8%
	Female	203	49.2%
Religion	Orthodox	126	30.5%
	Muslim	136	32.9%
	Protestant	102	24.7%
	Catholic	49	11.9%
Profession	Medical Doctor	123	29.8%
	Midwife	172	41.6%
	Nurse	44	10.7%
	Health officer	21	5.1%
	Anesthetist	35	8.5%
	Others*	18	4.4%
Experience	<= 5	276	66.8%
	6–9	81	19.6%
	>= 10	56	13.6%
Level of Hospital	Comprehensive	82	19.9%
	General	169	40.9%
	Primary	162	39.2%
Type of Hospital	Public	288	69.7%
	Private	125	30.3%

Others* Anesthesiologist, Integrated Emergency Surgical Officers

### Individual, facility, and client-related characteristics

About 52.1% of the respondents had adequate knowledge of obstetric analgesia methods for labor pain management. Among the methods, 88.9% and 66.3% of the respondents knew NSAID and epidural analgesia as pharmacologic method, while 88.6% and 72.9% of them knew back massage and Psychotherapy from non-pharmacologic methods for labor pain management. The findings of the study revealed that 60.8% of the participants had a favorable attitude toward the use of obstetric analgesia for labor pain management. Among the total participants, 67.8% confirmed the availability of analgesic drugs in their facilities, and 51.8% had never asked a laboring mother to provide pain relief (Table 2).

**Table 2:** Individual, facility, and client related characteristics of obstetric care providers working in Hospitals of West Arsi and Arsi zone, Oromia region, Ethiopia, 2023

Variables	Category	Frequency (%)
Qualification	Diploma	31 (7.5)
	BSc	208 (50.4)
	Masters	52 (12.6)
	General Practitioner	71 (17.2)
	Obstetrician	34(8.2)
	Resident	17(4.1)
Knowledge	Inadequate knowledge	198 (47.9)
	Adequate Knowledge	215 (52.1)
Attitude	Unfavorable Attitude	162 (39.2)
	Favorable Attitude	251 (60.8)
Perception of Labor pain	Mild to moderate	19 (4.6)
	Severe pain	394 (95.4)
Best method	Pharmacologic Method	185 (44.8)
	Nonpharmacologic Method	207 (50.1)
Heard of the WHO pain ladder	Yes	277 (67.1)
	No	136 (32.9)
Training	Yes	72 (17.4)
	No	341 (82.6)
Availability of drug	Yes	280(67.8)
	No	133(32.2)
Type of Available drug	Pethidine	191(46.2)
	Diclofenac	117(42.9)
	Paracetamol	117(28.3)
	Regional	90(21.8)
	Hyoscine	31(7.5)
Analgesic free of charge	Yes	193(46.7)
	No	87(21.1)
Place of storage	Drug store	113(27.4)
	Dispensary	152(36.8)
	Labor Ward	15(3.6)
Guideline and Protocol	Yes	122(29.5)
	No	291(70.5)
Perception of Adequate Staff	Yes	124(30.0)
	No	289(70.1)
Encountered Maternal refusal	Yes	58(14.0)
	No	142(34.4)

### Utilization of obstetric analgesia

Of the 413 obstetric care providers who participated in this study, 52.3% reported practicing labor pain management methods in the previous month. Only 39.8% provided labor pain management methods on a routine basis. Regarding labor pain management methods, 21.5% of the participants used non-pharmacologic methods, 16.2% used pharmacologic methods, and 14.5% used both methods in the previous month. Furthermore, 23.5% of participants preferred non-pharmacologic methods. The unavailability of drugs (44.0%), high patient flow (27.9%), and lack of knowledge (16.4%) were some of the reasons mentioned by participants for not utilizing obstetric analgesia. Meanwhile, 13.7% of the participants responded that they had no reason for not practising the methods.

### Factors associated with the utilization of obstetric analgesia

Among the candidate variables included in the multivariable logistic regression model, four factor (sex of respondents, level of hospital, knowledge, and Attitude) were found to be significantly associated with the utilization of obstetric analgesia.

Female professionals had a 1.86 times higher likelihood of using obstetric analgesia methods (AOR: 1.86, 95% CI: 1.14, 3.04). Obstetric care providers working in general and primary hospitals were 2.86 times more likely to use obstetric analgesia (AOR: 2.86, 95% CI: 1.41, 5.80), while those working in comprehensive and specialized hospitals were 3.89 times more likely to use obstetric analgesia (AOR: 3.89, 95% CI: 1.85, 8.17).

Obstetric care providers with adequate knowledge of obstetric analgesia methods were 1.94 times more likely to use obstetric analgesia than those with inadequate knowledge [AOR: 1.94(95% CI: 1.05, 3.57)]. Similarly, obstetric care providers who have a favorable attitude towards using obstetric analgesia were 6.92 times more likely to use obstetric analgesia than their counterparts [AOR: 6.92(95% CI: 3.97, 12.06)] (Table 3).

**Table 3:** Multivariable logistic regression analysis of factors associated with utilization of obstetric analgesia among obstetric care providers working in hospitals in the West Arsi and Arsi Zones, Oromia region, Ethiopia, 2023

Variables	Utilization		COR (95%CI)	*P* value	AOR (95%CI)
	Yes	No			
Sex					
Male	93	117	1	1	1
Female	123	80	1.93(1.31–2.86)	0.013	1.86(1.14–3.04) *
Profession					
Medical Doctor	55	68	0.30(0.11–0.83)	0.40	0.61(0.19–1.94)
Midwife	93	79	0.42(0.16–1.13)	0.32	1.86(0.55–6.26)
Nurse	15	29	0.19(0.06–0.59)	0.79	1.22(0.29–5.09)
Health officer	14	7	0.75(0.20–2.77)	0.32	2.17(0.48–9.93)
Anesthetist	27	8	1.27(0.37–4.31)	0.66	1.38(0.32–5.98)
Others*	12	6	1	1	1
Religion					
Orthodox	70	56	1.20(0.62–2.33)	0.18	1.73(0.78–3.83)
Muslim	60	76	0.76(0.39–1.46)	0.67	0.84(0.38–1.84)
Protestant	61	41	1.43(0.72–2.84)	0.08	2.12(0.92–4.87)
Catholic	25	24	1	1	1
Experience					
<= 5	143	133	1	1	1
6–9	43	38	1.05(0.64–1.73)	0.98	0.99(0.54–1.83)
>= 10	30	26	1.07(0.60–1.91)	0.55	1.24(0.61–2.52)
Level of Hospital					
Comprehensive	27	55	1	1	1
General	92	77	2.43(1.40–4.22)	0.004	2.86(1.41–5.80) *
Primary	97	65	3.04(1.74–5.31)	0.000	3.89(1.85–8.17) *
Knowledge					
Inadequate	90	108	1	1	1
Adequate	126	89	1.69(1.15–2.51)	0.034	1.94(1.05–3.57) *
Type of Hospital					
Public	155	133	1	1	1
Private	61	64	0.82(0.54–1.25)	0.30	0.75(0.44–1.29)
Attitude					
Unfavorable	42	120	1	1	1
Favorable	174	77	6.45(4.15–10.05)	0.0001	6.92(3.97–12.06) *
Maternal refusal					
Yes	25	33	1	1	1
No	98	44	2.94(1.56–5.52)	0.90	1.93(0.90–4.14)

Others* Anesthesiologist, Integrated Emergency Surgical Officers, * statistically significant at p-value < 0.05

## Discussion

A total of 413 obstetric care providers participated in this study. Among them, 52.3% reported using obstetric analgesia methods to manage labor pain in the previous month. The utilization of labor pain management methods was significantly associated with factors such as being female, hospital level, knowledge, and attitude of obstetric care providers. However, it is important to note that this proportion is insufficient to meet the needs of women seeking pain relief during labor and delivery.

The findings of this study are consistent with those of studies conducted in Belgium (47.8%), Nigeria (48.4%), Eastern Ethiopia (50.9%), Debre Markos (49.1%), and East Gojjam (48.9%) [7, 20, 23–25]. However, the utilization in this study was lower than that in studies conducted in Australia (68.4%), Norway (75%), and Kenya (61.5%) [26–28]. This discrepancy could be due to differences in the advancement of the healthcare system, availability of analgesic drugs and materials, and increased awareness of the use of obstetric analgesia among obstetric care providers.

Moreover, the findings of this study were higher than those of studies conducted in southern Ethiopia (37.9%), Amhara Referral Hospital (40.1%), West Shewa (46%), and Addis Ababa (36.6%) [19, 21, 29, 30]. This variation might be due to the difference in sample size and the time difference related to previous studies. Additionally, the current study involved only healthcare providers working at the hospital level, where the possibility of obtaining highly qualified professionals was high.

In this study, female obstetric care providers were 1.86 times more likely to practice labor pain management methods than their male counterparts. This finding aligns with those of a study conducted in Nigeria, Harari, and eastern Ethiopia, where female providers were more likely to provide obstetric analgesia [23, 31, 32]. The justification for this is that female professionals, having likely experienced childbirth themselves, can more readily relate to the pain of labor and have greater empathy for other women.

According to the findings of this study, obstetric care providers with adequate knowledge of labor pain management methods were 1.94 times more likely to use obstetric analgesia. This finding is consistent with that of a study conducted in Zaria Nigeria, Addis Ababa, and Harari [7, 21, 31]. A possible justification for this is that having comprehensive knowledge of different labor pain management methods allows obstetric care providers to make informed decisions and practice appropriate labor management methods.

However, these findings conflict with those of a study conducted in Kembata and the Amhara regional state, which found that inadequate knowledge was an influencing factor in the use of obstetric analgesia [29, 30]. This might be due to individual differences in the awareness of obstetric analgesia methods and the fact that the average score of each participant determined the level of knowledge in the study.

Having a favorable attitude toward using obstetric analgesia was found to be associated with the provision of obstetric analgesia, as obstetric care providers with favorable attitudes were 6.92 times more likely to provide obstetric analgesia. This finding is similar to those of studies conducted in central Ethiopia, Harari, Hawasa City, Kembata Tembaro, and Tigray General Hospital, where a favorable attitude was found to be an indicator of the utilization of obstetric analgesia [19, 29, 31, 33, 34]. This could be because those obstetric care providers with favorable attitudes are aware of the severity and pain experienced by laboring women and are knowledgeable about the safety and efficacy of different methods, which allows them to provide the appropriate method.

The findings of this study revealed that professionals working in general and primary hospitals were 2.86 and 3.89 times more likely to use obstetric analgesia, respectively. This finding conflicts with a study conducted in Nigeria, where obstetricians working in tertiary hospitals were more likely to use epidural analgesics [32]. This variation might be because these hospitals specialize in managing complicated and high risk cases, and may focus on managing complicated conditions, and providing routine labor and delivery care may not be prioritized in these settings.

### Limitation of this study

The limitation of this study is that it did not directly observe obstetric care providers while they were practicing the methods. Furthermore, the study did not compare variations between public and private hospitals.

## Conclusion

The findings of this study indicate that the utilization of obstetric analgesia is low compared to previous studies. According to the findings, obstetric care providers’ utilization of obstetric analgesia was influenced by having adequate knowledge, a favorable attitude, being a female professional, and the level of the hospital in which they work.

To improve the utilization of obstetric analgesia methods for labor pain management, we recommend that obstetric care providers be equipped with current knowledge of available obstetric analgesia method, their associated benefits, and risks. For researcher, we suggest conducting additional studies that integrate observation of obstetric care providers while they practice the method and entail a comparative study between private and public hospitals. Additionally, we recommend that a future study that include health centers are needed to provide a more comprehensive picture of the situation across all levels of healthcare facilities.

## Supplementary Information

Supplementary Material 1.

## Data Availability

The datasets used during the current study are available from the corresponding author upon reasonable request.

## Abbreviations

ACOG: American College of Obstetrics and Gynecology
AOR: Adjusted Odds Ratio
CI: Confidence Interval
COR: Crude Odds Ratio
IESO: Integrated Emergency Surgical officer
SPSS: Statistical Package for Social Science

## References

[1] Melzack R. Labour pain as a model of acute pain. In: pain. Elsevier Science; 1993. pp. 117–20. 10.1016/0304-3959(93)90071-V 8336982

[2] Whitburn LY. The nature of labour pain (Doctoral dissertation, La Trobe).

[3] Lauren Plante and Robert Gaiser. Practice Bulletin Obstetric Analgesia and Anesthesia. In: clinical management guidelines for obstetrician–gynecologists. 2019. pp. 208–25.

[4] Shnol H, Nicole Paul IB. Labor Pain Mechanisms. In. 2014. pp. 1–17. 10.1097/AIA.0000000000000019 24946040

[5] Mårtensson L, Bergh I. Effect of treatment for labor pain: Verbal reports versus visual analogue scale scores-A prospective randomized study. Int J Nurs Midwifery. 2011;3(4):43–7.

[6] Karn S, Yu H, Karna S, Chen L, Qiao D. Women ’ s Awareness and Attitudes towards Labor Analgesia Influencing Practice between Developed and Developing Countries. Adv Reprod Sci. 2016;(4):46–52.

[7] Ogboli-Nwasor E, Adaji SE, Bature SB, Shittu OS. Pain relief in labor: a survey of awareness, attitude, and practice of health care providers in Zaria, Nigeria. J Pain Res. 2011;17:227–32. 10.2147/JPR.S21085 PMC3160836 21887120

[8] Apfelbaum JL, Hawkins JL, Agarkar M, Bucklin BA, Connis RT, Gambling DR, et al. Practice guidelines for obstetric anesthesia: an updated report by the American Society of Anesthesiologists Task Force on obstetric anesthesia and the Society for Obstetric Anesthesia and Perinatology∗. Anesthesiology. 2016;124(2):270–300. 26580836 10.1097/ALN.0000000000000935

[9] Kenyon S, Ullman R, Mori R, Whittle M. Care of healthy women and their babies during childbirth: summary of NICE guidance. BMJ. 2007;335(7621):667–8. 10.1136/bmj.39322.703380.AD PMC1995472 17901518

[10] Food. Medicine and Healthcare Administration and Control Authority of Ethiopia Standard Treatment Guidelines Good Prescribing & for Better Health. 2014.

[11] Caton D, Corry MP, Frigoletto FD, Hopkins DP, Lieberman E, Mayberry L, et al. The nature and management of labor pain: Executive summary. Am J Obstet Gynecol. 2002;186(5 SUPPL):1–15. 12011869 10.1016/S0002-9378(02)70178-6

[12] Lundgren I, Dahlberg K. Women ’ s experience of pain during childbirth. Midwifery. 1995;14:105–10. 10.1016/s0266-6138(98)90007-9 10382479

[13] Baker A, Ferguson SA, Roach GD, Dawson D. Perceptions of labour pain by mothers and their attending midwives. J Adv Nurs. 2001;35(2):171–9. 11442696 10.1046/j.1365-2648.2001.01834.x

[14] GK P, Sameera L. Awareness of labour analgesia among antenatal women in semi urban area. Int J Reprod Contracept Obstet Gynecol. 2019;5(8):2613.

[15] Ebirim LN, Buowari OY, Ghosh S. Physical and psychological aspects of pain in obstetrics. Pain in perspective. IntechOpen; 2012.

[16] Ryding EL. Investigation of 33 women who demanded a cesarean section for personal reasons. Acta Obstet Gynecol Scand. 1993;72(4):280–5. 8389515 10.3109/00016349309068038

[17] Morgan BM, Bulpitt CJ, Clifton P, Lewis PJ. Analgesia and satisfaction in childbirth (The Queen Charlotte’s 1000 Mother survey). Lancet. 1982;320(8302):808–10. 10.1016/s0140-6736(82)92691-5 6126674

[18] Federal Ministry of Health (FMoH). Standard of Midwifery Care practice in Ethiopia. Addis Ababa (Ethiopia): FMoH, 2013.

[19] Terfasa EA, Bulto GA, Irenso DY. Obstetric analgesia utilization in labor pain management and associated factors among obstetric care providers in the West Shewa Zone, Central Ethiopia. SAGE Open Med. 2022;10:205031212210887. 10.1177/20503121221088705 PMC8943529 35342628

[20] Shiferaw A, Temesgen B, Alamirew NM, Wube T, Worku Y. Utilization of labor pain management methods and associated factors among obstetric care givers at public health institutions of East Gojjam Zone, Amhara region, Ethiopia, 2020: a facility based cross–sectional study. BMC Pregnancy Childbirth. 2022;22(1):803. 36319950 10.1186/s12884-022-05094-z PMC9623903

[21] Gido R, Yadeta TA, Tura AK. Utilization of obstetric analgesia for labor pain management and associated factors among obstetric care providers in public hospitals of Addis Ababa, Ethiopia: a cross‐sectional study. Obstet Gynecol Int. 2021;2021(1):9973001. 10.1155/2021/9973001 PMC8629664 34853595

[22] Wassihun B, Alemayehu Y, Gultie T, Tekabe B, Gebeyehu B. Non-pharmacological labor pain management practice and associated factors among skilled attendants working in public health facilities in Gamo and Gofa zone, Southern Ethiopia : A cross-sectional study. 2022;1–11. Available from: 10.1371/journal.pone.0266322. 10.1371/journal.pone.0266322 PMC9022872 35446867

[23] Eyeberu A, Getachew T, Debella A, Balis B, Eshetu B, Mesfin S, Bekele H, Tamiru D, Tiruye G, Degefa M, Alemu A. Utilization of pharmacological labour analgesia: a survey of obstetric care providers in eastern Ethiopia. Int Health. 2023;15(3):335–41. 10.1093/inthealth/ihac061 PMC10153555 36088530

[24] Christiaens W, Verhaeghe M, Bracke P. Pain acceptance and personal control in pain relief in two maternity care models: a cross-national comparison of Belgium and the Netherlands. BMC Health Serv Res. 2010;10(1):268. 10.1186/1472-6963-10-268 PMC2944275 20831798

[25] Melesse AS, Bayable SD, Ashebir YG, Ayenew NT, Fetene MB. Survey on knowledge, attitude and practice of labor analgesia among health care providers at Debre Markos Comprehensive Specialized Hospital, Ethiopia 2021. A cross-sectional study. Ann Med Surg. 2022;74:103306. 10.1016/j.amsu.2022.103306 PMC8818902 35145674

[26] Tveit TO, Halvorsen A, Rosland JH. Analgesia for labour: A survey of Norwegian practice - With a focus on parenteral opioids. Acta Anaesthesiol Scand. 2009;53(6):794–9. 19456300 10.1111/j.1399-6576.2009.01988.x

[27] Ouma Gabriel O, Orango E, Were K, Omwodo. Provision of labour analgesia and its related barriers among maternal health care providers in Kenya: An institution-based descriptive survey. 2022. pp. 1–21. Available from: 10.21203/rs.3.rs-1680097/v2.

[28] Steel A, Adams J, Sibbritt D, Broom A, Gallois C, Frawley J. Managing the pain of labour: Factors associated with the use of labour pain management for pregnant Australian women. Heal Expect. 2015;18(5):1633–44. 10.1111/hex.12155 PMC5060859 24304970

[29] Geltore TE, Taye A, Kelbore AG. Utilization of obstetric analgesia in labor pain management and associated factors among obstetric caregivers in public health facilities of Kembata Tembaro Zone, Southern Ethiopia. J Pain Res. 2018;11:3089–97. 30584351 10.2147/JPR.S165417 PMC6287531

[30] Bitew A, Workie A, Seyum T, Demeke T. Utilization of obstetric analgesia in labor pain management and associated factors among obstetric care givers in Amhara Regional State Referral Hospitals, Northwest Ethiopia: a hospital-based cross-sectional study. J Biomed Sci. 2016;5(2):3.

[31] Eyeberu A, Debela A, Getachew T, Dheresa M, Alemu A, Dessie Y. Obstetrics care providers attitude and utilization of non-pharmacological labor pain management in Harari regional state health facilities, Ethiopia. BMC Pregnancy Childbirth. 2022;22(1):1–9. Available from: 10.1186/s12884-022-04717-9. 10.1186/s12884-022-04717-9 PMC9066716 35509044

[32] Anozie OB, Lawani LO, Mamah JE, Esike CO, Ezeonu OP, Eze JN et al. Epidural analgesia for management of labour pain: Determinants and deterrents among obstetricians in Nigeria. Int J Women’s Heal Reprod Sci. 2018;6(4):410–4. Available from: 10.15296/ijwhr.2018.68.

[33] Wakgari N, Mekonnen M, Lema B, Negasu A, Lulu B, Abebe E. Labour pain management practices among obstetric care providers in Hawassa city, Ethiopia. Afr J Midwifery Womens Health. 2020;14(2):1–12.

[34] Sahile E, Yemaneh Y, Alehegn A, Nigussie W, Salahuddin M, Yekoye A, Gebeyehu N. Practice of labour pain management methods and associated factors among skilled attendants working at general hospitals in Tigray Region, North Ethiopia: hospital based cross-sectional study design. Health Sci J. 2017;11(4):516.

