# Supplementary material for: Utilization of obstetric analgesia for labor pain management and associated factors among obstetric care providers working in hospitals of South East and South Central Ethiopia, 2023
Source: BMC Pregnancy Childbirth. 2026 May 22;26:773. doi: 10.1186/s12884-026-09310-y (PMC13371265; doi:10.1186/s12884-026-09310-y)
Supplement: Supplementary file 1 — Supplementary Material 1. [file 12884_2026_9310_MOESM1_ESM.docx]

**ANNEXES**

**Annexes I: Participant Information Sheet**

My name is ___________________I am working as a data collector for the study being conducted in this hospital. I kindly request you to offer me your attention to explain to you about the study you are being selected as the study participant.

**The study Title**: - Utilization of obstetric Analgesia for labour pain management and associated factors among obstetric Care providers in the Hospitals of West Arsi and Arsi zone, Oromia region, 2023.

**Purpose/aim of study**: To assess utilization of obstetric Analgesia for labour pain management and associated factors that affect the implementation of effective utilization of obstetrics analgesia among obstetrics care giver in the Hospitals of West Arsi and Arsi zone, Oromia region, 2023. It can also provide important information to program managers to address such issues in the future.

**Procedure and duration**: I will give you a self-administered questionnaire to provide me with pertinent data that is helpful for the study. There are 42 questions. The questionnaire will be filled by you. The questionnaire will take about 30 minutes, so I kindly request you to spare this desired time to fill the questioner.

**Risks and benefits**: The risk of participating in this study is minimal risk at all, but only taking few minutes from your time. There would not be any direct payment for participating in this study. But the findings from this research may reveal important information for the Hospitals and local health care planners.

**Confidentiality**: The information that you will provide will be kept confidential. There will be no information that will identify you in particular. The questionnaire will be coded to exclude showing names. No reference will be made in oral or written reports that could link participants to the research.

**Rights:** Participation for this study is fully voluntary. You have the right to declare to participate or not to participate in this study. If you decide to terminate participation in the study, you have the right to withdraw from the study at any time and this will not label you for any loss of benefits which you otherwise will be entitled.

**Annexes II: English Version Questionnaire**

Participant Code: _____________________

Code of the hospital: __________________

**Instructions:**

- These questionnaires are designed to collect information through self-administered questioner from our study participants.
- Please Encircle from the given option and write in the space provided for a question which has no option.

| **NO** | **Question** | **Response** | **Skip to** |
| --- | --- | --- | --- |
| **Part I:- Socio-demographic characteristics** | | | |
| **101** | Age in years | **_____________** |  |
| **102** | Sex | 1. Male  2. Female |  |
| **103** | Religion | 1. Orthodox  2. Muslim  3. Protestant  4. Catholic  5. others______ |  |
| **104** | Profession | 1.Medical doctor  2.Midwife  3.Nurse  4. IESO  5. Health officers  6. Anestheologist  7. Anesthetist |  |
| **105** | Clinical Experience in years | **___________** |  |
| **106** | What is the level of the Hospital you are practicing? | 1.Comprehensive and specialized hospital  2. General Hospital  3. Primary Hospital |  |
| **107** | Type of Hospital? | 1. Public/Government  2. Private |  |
| **Part II: Individual Related Questions** | | | |
| **201** | Qualification | 1.Diploma  2.BSC  3.Masters  4.General practitioner  5.Obstetrician/Gynecologist  6.Resident doctor  7.Other______________ |  |
| **Pain Perception Question** | | | |
| **202** | What is your perception of labour pain? | 1. Mild pain  2. Moderate pain  3. Severe pain |  |
|  | **Knowledge Related Questions** | |  |
| **203** | Do you know about labour pain management methods? | 1.Yes  2.No |  |
| **204** | Which of Pharmacologic method of labour pain management do you know? | 1. Systemic opioids  2. NSAID  3. Epidural Analgesia  4. Inhalation |  |
| **205** | Which of non-pharmacologic labour pain management do you know? | 1. Psychotherapy 2. Allow the mother to Ambulate 3. Massage the back 4. Allow free vertical positioning 5. Transcutaneous electrical nerve stimulation 6. Show the patient how to bear down 7. Acupuncture 8. Hypnosis 9. Allow companion of her Choice 10. Music therapy |  |
| **206** | If you know about labor analgesia, do obstetrics analgesia have side effect on labor and delivery outcome? | 1.Yes  2. No | If no skip to 208 |
| **207** | If” yes” to question 206, what is the side effect of analgesia on labour and its outcome? More than one answer is possible | 1. Delay progress of labour  2. Cause fetal distress  3. Increase instrumental Delivery  4. Increase C/S delivery  5. If other specify______. |  |
| **208** | Which type is best for labour pain management? | 1. Pharmacological method  2. Non-pharmacological method |  |
| **209** | Have you heard about WHO pain ladder? | 1.Yes  2. No |  |
|  | **Attitude Related Questions** | |  |
| **301** | Do you believe labour pain management methods can alleviate or help the mother to cope labour pain? | 1. Strongly disagree  2. Disagree  3.Undecided  4.Agree  5.Strongly agree |  |
| **302** | Do you think every mother during labour should be managed? | 1. Strongly disagree  2. Disagree  3.Undecided  4.Agree  5.Strongly agree |  |
| **303** | Do you think women should not have to suffer the natural labor pain? | 1. Strongly disagree  2. Disagree  3.Undecided  4.Agree  5.Strongly agree |  |
| **304** | Do you think analgesic is necessary for managing labour pain? | 1. Strongly disagree  2. Disagree  3.Undecided  4.Agree  5.Strongly agree |  |
| **305** | Do you believe that you have responsibility and obligation to manage labour pain? | 1. Strongly disagree  2. Disagree  3.Undecided  4.Agree  5.Strongly agree |  |
| **306** | Do you think use of analgesia can influence the progress of labor? | 1. Strongly disagree  2. Disagree  3.Undecided  4.Agree  5.Strongly agree |  |
| **307** | Do you think use of labor analgesia causes late presentation? | 1. Strongly disagree  2. Disagree  3.Undecided  4.Agree  5.Strongly agree |  |
| **308** | Do you think use of labor analgesia can cause fetal distress? | 1. Strongly disagree  2. Disagree  3.Undecided  4.Agree  5.Strongly agree |  |
| **309** | Do you think labor analgesia offers a better birth experience? | 1. Strongly disagree  2. Disagree  3.Undecided  4.Agree  5.Strongly agree |  |
| **310** | Do you think labor pain relief services should include awareness creation and education for client? | 1. Strongly disagree  2. Disagree  3.Undecided  4.Agree  5.Strongly agree |  |
|  | | | |
| **401** | Have you got any special training for managing labour pain? | 1. Yes  2. No |  |
| **402** | Do you allow a laboring mother to have a companion of her choice during labour and delivery? | 1.Yes  2. No |  |
| **Part III: Use of obstetric Analgesia Related Question** | | | |
| **501** | Have you ever provided any labour pain relief method in the past one month? | 1.Yes  2. No | If no skip to 507 |
| **502** | If” yes” to question No 501, which method did you provided? | 1. Pharmacological  2. Non-pharmacological  3. Both |  |
| **503** | If “Pharmacological” for question 502 which method? You can answer more than one choice | 1. Pethidine  2.Tramadol  3. Diclofenac  4. Paracetamol  5. Hyoscine  6.Epidural  7.Entonoux(N_2_O) |  |
| **504** | If “non-pharmacological” to question 502, which type of non-pharmacologic? You can answer more than one | 1. Psychotherapy 2. Allow the mother to Ambulate 3. Massage the back 4. Allow free vertical Positioning 5. Show the patient how to bear down 6. Hot compress 7. Music therapy 8. If other specify_______. |  |
| **505** | What is the pattern/frequency of your labor analgesia usage? | 1.Routinely  2.Somettimes  3.on maternal request |  |
| **506** | Which method do you prefer for managing labour pain? | 1. pharmacologic method  2. Non-pharmacologic method  3. Both |  |
| **507** | If “No” for question 501 what is the reasons for your non-utilization of labour pain management methods? Multiple options are possible…. | 1. Lack of knowledge  2.Drug unavailable  3. No equipment  4. Lack of skills  5.Managerial problems  6. Culture /religion  7. Maternal Refusal  8. High patient flow  9. No reason  10. Other_________ |  |
| **Part IV: Facility Related Question** | | | |
| **601** | Is there labour pain managing drugs/ analgesics available at your hospital? | 1.Yes  2. No | If no skip to 605 |
| **602** | If “yes’’ to question No 701, which type? You can answer more than one | 1.Pethidine  2. Diclofenac  3. Paracetamol  4. Hyoscine  5.Tramadol  6.Regional analgesics  7. If other specify_________ |  |
| **603** | If, yes where is the place of storage? | 1.Drug store  2.Dispensary  3.Labour ward |  |
| **604** | Are obstetric labour analgesia drugs free of charge in your facility? | 1.Yes  2.No |  |
| **605** | Are there guidelines and protocols about provision of pharmacologic and non-pharmacologic labour analgesia? | 1.Yes  2.No |  |
| **606** | Are there adequate number of staff in your Hospital? | 1.Yes  2.No |  |
| **Part V: Client related Question** | | | |
| **701** | Have you ever asked laboring mother to provide labour pain relief method? | 1.Yes  2.No |  |
| **702** | If yes to no 701, have you ever experienced a refusal from the mother for any of labour pain relief method? | 1.Yes  2.No |  |

**Thank you for your participation!!!**
